# Molecular Genetic Dissection of Inflammatory Linear Verrucous Epidermal Naevus Leads to Successful Targeted Therapy

**DOI:** 10.1016/j.jid.2021.02.765

**Published:** 2021-12

**Authors:** Melissa Riachi, Satyamaanasa Polubothu, Paulina Stadnik, Connor Hughes, Sara Barberan Martin, Carolyn R. Charman, Iek Leng Cheng, Karolina Gholam, Olumide Ogunbiyi, David G. Paige, Neil J. Sebire, Alan Pittman, Wei-Li Di, Veronica A. Kinsler

**Affiliations:** 1 Genetics and Genomic Medicine, University College London Great Ormond Street Institute of Child Health, London, United Kingdom; 2 Mosaicism and Precision Medicine Laboratory, The Francis Crick Institute, London, United Kingdom; 3 Paediatric Dermatology, Great Ormond Street Hospital for Children, London, United Kingdom; 4 Dermatology, Royal Devon and Exeter Hospital, Exeter, United Kingdom; 5 Pharmacy, Great Ormond Street Hospital for Children, London, United Kingdom; 6 Paediatric Pathology, Department of Histopathology, Great Ormond Street Hospital for Children, London, United Kingdom; 7 Dermatology, Royal London Hospital, London, United Kingdom; 8 Bioinformatics, St George’s University of London, London, United Kingdom; 9 Immunobiology Section, Infection, Immunity and Inflammation Programme, University College London Great Ormond Street Institute of Child Health, London, United Kingdom

**Keywords:** ILVEN, inflammatory linear verrucous epidermal naevus, KC, keratinocyte

To the Editor

Inflammatory linear verrucous epidermal naevus (ILVEN) is a rare skin condition. Classically, it presents at birth or within the first year of life, frequently progressing during early childhood. Diagnostic criteria are erythematous verrucous hyperkeratosis in a fine and whorled Blaschko-linear pattern, intense pruritus, early age of onset, histological features, and resistance to treatment (Morag and Metzker, 1985). The cause of ILVEN has been unknown; however, a single case of mosaicism in gene *GJA1* has recently been reported (Umegaki-Arao et al., 2017). We sought to investigate the genetics of ILVEN with a view to new therapeutic angles.

A total of 15 children with ILVEN and six normal controls (from surgery where excess normal skin was available) were recruited with written informed consent by their parents or guardians and Research Ethics Committee approval from the Great Ormond Street Hospital Research and Development office. The patients’ parents/guardians consented to the publication of the patients' images. DNA and RNA were extracted from skin biopsies of the affected tissue, DNA was extracted from blood by standard methods and affected skin keratinocytes (KCs) were cultured and immortalized where possible (Lenti-HPV-16 E6/E7 Virus). Deep whole-exome sequencing of blood and affected skin was performed on patient samples, and data were analyzed using an optimized bioinformatic pathway for the detection of low-level somatic variants as previously published (Al-Olabi et al., 2018). Pathogenic *GJA1* variants were not found in any patient. The clinical and histological features of patients 1 and 2 are shown in Figures 1 and 2a and b and Supplementary Table S1.

**Figure 1:**
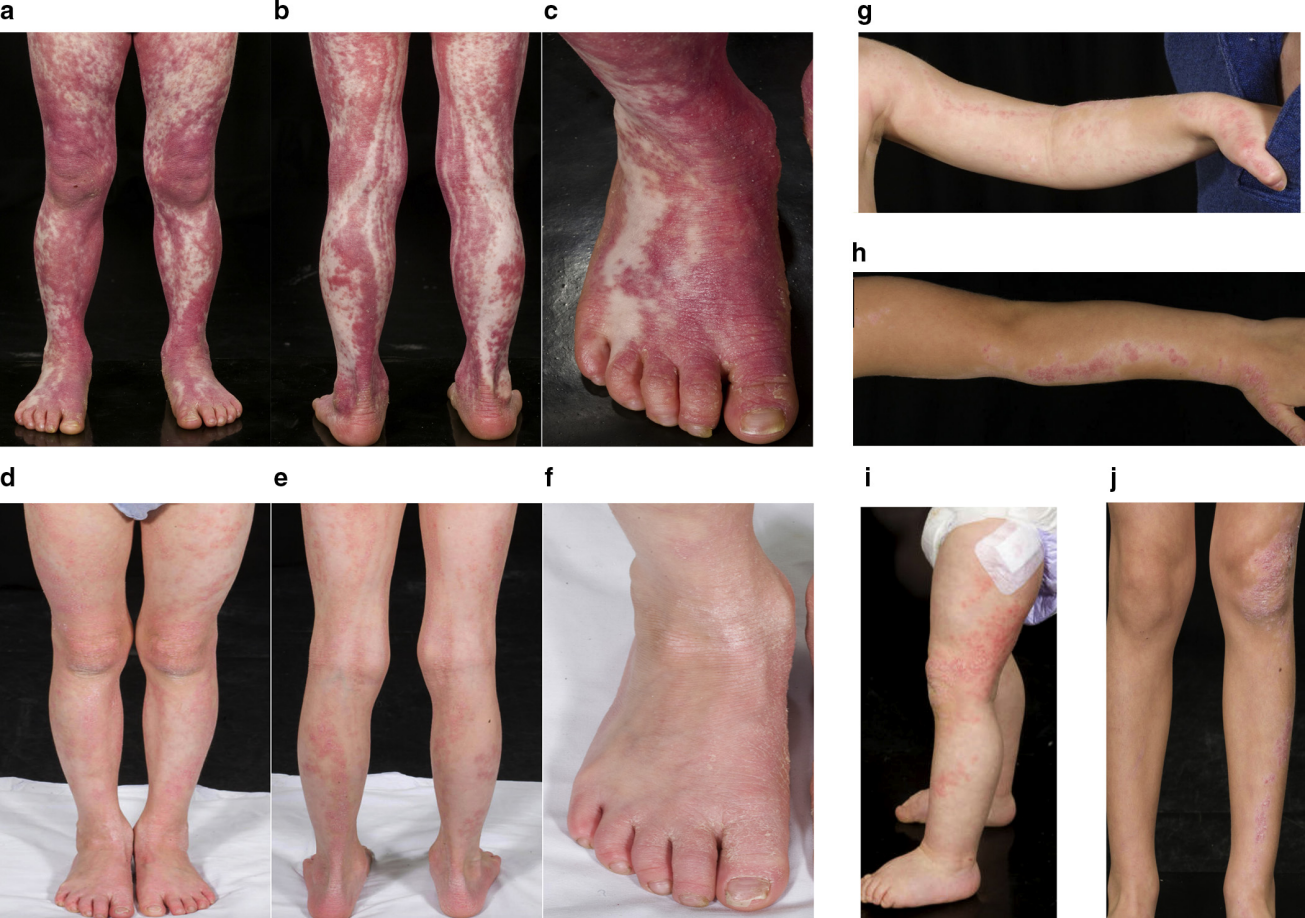
**Clinical features of *CARD14* mosaic ILVEN and dramatic response to targeted therapy in one patient.** Patient 1 pre-treatment (**a–c**) and 3 months post commencing Ustekinumab (**d–f**), showing dramatic reduction in erythema and hyperkeratosis. Patient 2 pre-treatment showing predominantly left-sided Blaschko-linear inflammatory and hyperkeratotic skin lesions at 1 year (**g,i**) and 4 years (**h,j**). The patients’ parents/guardians consented to the publication of the patients' images.

**Figure 2:**
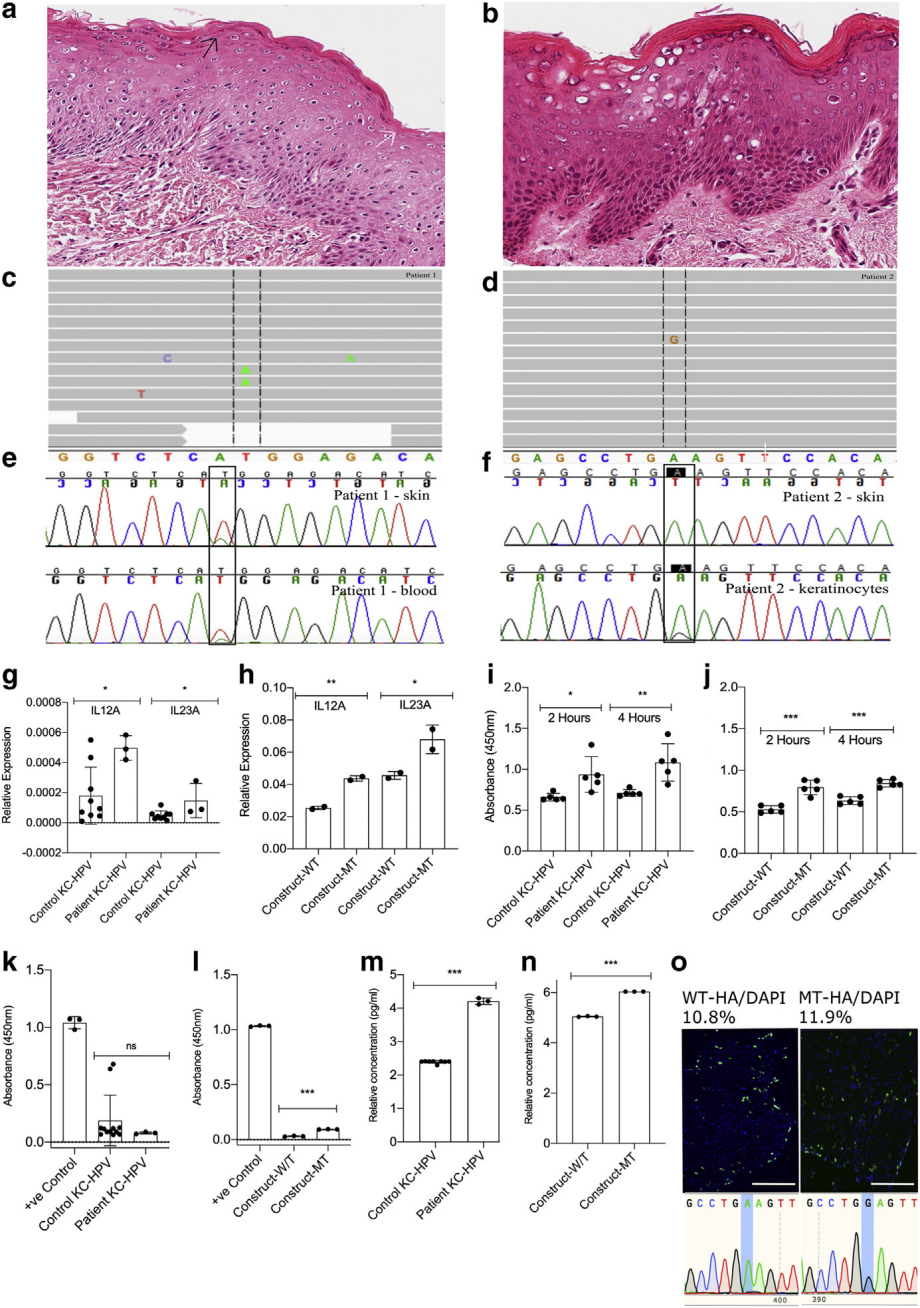
**Histological features and mosaic genetic variants in *CARD14* ILVEN.** (**a, c, e**) Patient 1 and (**b, d, f, g, h, i, j, k, l, m, n**) patient 2. **(a, b)** Histology demonstrating alternating orthokeratosis (white arrow) and parakeratosis (black arrow) in patient 1, with generalized disruption of cornification in patient 2. Histological variability between ILVEN samples (from clinical diagnosis) was found to be very broad. **(c, d)** Whole-exome sequencing visualized in the Integrative Genomics Viewer (Broad Institute, Cambridge, MA) shows mosaic *CARD14* missense variants c. 356T > A, p. (M119K) (for patient 1 in **c**) and c.277A > G, p.(K93E) (for patient 2 in **d**). **(e, f)** Sanger sequencing chromatograms confirm the variants. Cultured patient KCs and SVK_14_ cells transfected with a mutant *CARD14* construct express increased *IL12* and *IL23* at mRNA and protein level, proliferate faster than controls, and show variable activity of NF-κB p65. (**g, h**) QRT-PCR demonstrating a significant increase in IL-12A and IL-23A in cultured KCs from the affected skin from patient 2 and in SVK_14_ cells transfected with the mutant *CARD14* construct in comparison to control patient KCs (n = 3) and SVK_14_ cells transfected with the wild-type *CARD14* construct, respectively. Mean relative gene expression of five replicates per patient sample and duplicates per SVK_14_ sample was calculated with SD. **(i, j)** WST-1 proliferation assay showing a proliferation increase in KCs cultured from patient 2 and in SVK_14_ cells transfected with the mutant *CARD14* construct compared to control patient KCs (n = 3) and SVK_14_ cells transfected with the wild-type *CARD14* construct, respectively, measured at 450 nm after 2 and 4 hours. The KCs were cultured for 8 days before proliferation measurement. The mean absorbance of five replicates is shown with SD. **(k)** Nuclear extracts from patient 2 KCs do not show a difference in NF-κB p65 activity when compared to control patient KCs (n = 6). **(l)** Nuclear extracts from SVK_14_ cells transfected with the mutant *CARD14* construct show an increase in NF-κB p65 activity when compared with SVK_14_ cells transfected with the wild-type *CARD14* construct. The mean absorbance of triplicates for patient/control KCs and positive control is shown with SD. **(m, n)** Patient 2 KCs and SVK_14_ cells transfected with the mutant *CARD14* construct have significantly increased levels of IL-12 and IL-23 secreted in the supernatant compared to control KC cell lines (n = 4) and SVK_14_ cells transfected with the wild-type *CARD14* construct, respectively. The mean absorbance of triplicates is shown with SD. All *P*-values were calculated by Students *t*-test using Prism, version 7.0 (GraphPad Software, San Diego, CA). Asterisks indicate a *P*-value < 0.05. **(o)** Immunofluorescent anti-HA staining of SVK_14_ cells transfected with *CARD14* wild-type and mutant pcDNA3.1-HA constructs with Sanger-sequencing validation. Bar = 400 um. HA, hemagglutinin; ILVEN, inflammatory linear verrucous epidermal naevus; KC, keratinocyte; QRT-PCR, quantitative real-time reverse transcriptase‒PCR.

Heterozygous missense variants in gene *CARD14* were detected in 2 of 15 patients (Figure 2c and d). In both patients, the allelic load was compatible with that of a mosaic variant. In patient 1, the variant was present at 20% in both the blood and DNA extracted directly from a whole punch biopsy of the affected skin (c.356T > A, p. (M119K)); and in patient 2, it was present at 1% from DNA extracted directly from the epidermis of the affected skin and it was undetectable in the blood (4/313 reads in skin, c.277A>G, p.(K93E)). We had intended that whole-exome sequencing of the epidermis in patient 2 might have increased the mutant allele load; however, this was not the case, and the 1% load may have been due to mainly cornified epidermis being sequenced. However, both variants were convincing on whole-exome sequencing raw data, and both were clearly confirmed by Sanger sequencing (Figure 2e and f). The missense variant in patient 1 affects the same codon as one previously published in a non-mosaic state causing pityriasis rubra pilaris (Lwin et al., 2018), supporting its likely pathogenicity *in vivo* and also supported by *in silico* predictions (SIFT Tolerated, Polyphen2 Benign, Mutation Taster Disease Causing, PROVEAN Neutral, CONDEL Neutral, combined annotation‒dependent depletion score 22.6). The variant in patient 2 is predicted overall likely pathogenic *in silico* (SIFT Tolerated, Polyphen2 Probably Damaging, Mutation Taster Disease Causing, PROVEAN Neutral, CONDEL Deleterious, combined annotation‒dependent depletion score 24.1), and since it was to our knowledge previously unreported, we went on to characterize its functional effects. Cultured patient KCs from patient 2 were used to model the variant in the most biologically similar manner. In addition, the patient 2 variant was modeled in a KC cell line (SVK_14_) that was transfected (Lipofectamine 2000) with *CARD14* wild-type and mutant (c.277A > G) pcDNA3.1-HA constructs (Figure 2o). The culture of KCs from patient 1 unfortunately failed, and it was not deemed ethical to take further biopsies from a child for this purpose only.

Quantitative real-time reverse transcription–PCR showed a significant increase in IL-12A and IL-23A in cultured patient KCs and SVK_14_ cells transfected with the mutant *CARD14* construct compared to identically–handled KCs from grouped normal controls (Figure 2g) and SVK_14_ cells transfected with the wild-type *CARD14* construct (Figure 2h). This was further validated at the protein level by IL-12/IL-23 p40 ELISA (Figure 2m and n) (Invitrogen, Waltham, CA). In addition, WST-1 assay (Sigma-Aldrich, St. Louis, MO) showed a significant increase in proliferation rate in patient KCs and SVK_14_ cells transfected with the mutant *CARD14* construct (Figure 2i and j). A significant increase in NF-κB p65 subunit activity was shown by ELISA in nuclear extracts from SVK_14_ cells transfected with the mutant *CARD14* construct (Figure 2l) but not in patient KC nuclear extracts (Figure 2k) (Abcam, Cambridge, United Kingdom), potentially owing to the less physiological model of overexpression in the cell line model.

Inherited (nonmosaic) heterozygous mutations in *CARD14* were recently described as rare causes of psoriasis (Jordan et al., 2012) and pityriasis rubra pilaris (Fuchs-Telem et al., 2012). Variants affecting certain domains of *CARD14* were initially described as leading to the activation of NF-κB in the skin (Fuchs-Telem et al., 2012). However, differences between wild-type and variant *CARD14* effects on NF-κB are modest (Li et al., 2015), and not all pathogenic variants increase the activation of NF-κB (Bertin et al., 2001). This includes some of those located in the CARD domain (amino acid sequences 15‒107) (Israel and Mellett, 2018) such as that in patient 2. Treatment of patients with germline *CARD14* variants with Ustekinumab has been highly successful (Eytan et al., 2014; Lwin et al., 2018); however, direct measurement of the effect of *CARD14* variants on IL-12 and IL-23 expression has not previously been performed (Teng et al., 2015). Our findings suggest that IL-12 and IL-23 could be increased by *CARD14* variants in a non‒NF-κB‒dependent manner.

Patient 1 had been resistant to multiple therapies (cyclosporine, acitretin, oral prednisolone), and she had faltering growth (height and weight below the 0.4th centile by age 3 years; birth weight 50th–75th percentile). With hospital drug and therapeutics committee approval, we started treatment at the age of 6 years with Ustekinumab (0.75 mg/kg/ dose at 0 and 1 months and 3 monthly thereafter, as per psoriasis protocol). She has had a dramatic and sustained improvement in her skin, now 20 months into treatment, but has required an increase to 8-weekly dosing to maintain effect between doses. She also exhibited catch-up growth, with height and weight improving from the <0.4th to 2–9th percentile within 3 months (Figure 1d and f) and no adverse effects. Patient 2 is younger and less symptomatic (Figure 1g and j) and has not required treatment.

Historically, there has been debate about the clinical and histopathological similarities of ILVEN to congenital hemidysplasia with ichthyosiform erythroderma and limb defects syndrome and to psoriasis (Happle, 1991; Ito et al., 1991; Moss and Burn, 1990; Welch et al., 1993). We consider that these debates are likely the result of genetic heterogeneity in ILVEN and that the term ILVEN is a clinical description rather than a single histopathological or genetic entity.

We identify in this study that heterozygous missense variants in *CARD14* are a recurrent cause of this phenotype, leading to successful targeted medical therapy in one patient. Indications for treatment should be made on an individual patient basis. Genetic counseling should be considered in ILVEN as in these cases, it could be passed on as pityriasis rubra pilaris or psoriasis. These findings underline the power of molecular genetic characterization of rare diseases alongside clinical and histopathological phenotyping.

### Data availability statement

No datasets were generated or analyzed during this study.

## ORCIDs

Melissa Riachi: http://orcid.org/0000-0001-7278-1780

Satyamaanasa Polubothu: http://orcid.org/0000-0001-7195-5670

Paulina Stadnik: http://orcid.org/0000-0001-9711-4933

Hughes Connor: http://orcid.org/0000-0001-9456-1324

Sara Barberan Martin: http://orcid.org/0000-0003-0142-4078

Carolyn R. Charman: http://orcid.org/0000-0001-6652-7671

Iek Leng Cheng: http://orcid.org/0000-0003-1010-8085

Karolina Gholam: http://orcid.org/0000-0002-8109-6993

Olumide Ogunbiyi: http://orcid.org/0000-0001-5208-5526

David G. Paige: http://orcid.org/0000-0003-3583-020X

Neil J. Sebire: http://orcid.org/0000-0001-5348-9063

Alan Pittman: http://orcid.org/0000-0002-8112-2987

Wei-Li Di: http://orcid.org/0000-0002-4851-1649

Veronica A. Kinsler: http://orcid.org/0000-0001-6256-327X

## Conflict of Interest

The authors state no conflict of interest.

## Figures and Tables

**Supplementary Table S1:** Detailed Clinical Features of Patients 1 and 2

Clinical Features	Patient 1	Patient 2
Age of onset	11 mo	1 y
Lesion type	Blaschko-linear erythematous, hyperkeratotic, pruritic	Blaschko-linear erythematous, hyperkeratotic
Lesion distribution	Generalized	Appeared on left thumb at ages 4‒6 wk
Lesion extent	Facial, truncal, and all limbs	Facial, truncal, all limbs
Unilateral / Bilateral	Bilateral	Initially unilateral on the left side but progressed to bilateral
Palmoplantar involvement (Y/N) and which type	Diffuse palmoplantar keratoderma	Linear palmoplantar keratoderma in continuity with arm lesions

Abbreviations: N, no; Y, yes.
